# Integrative Bioinformatics Analysis Demonstrates the Prognostic Value of Chromatin Accessibility Biomarkers in Clear Cell Renal Cell Carcinoma

**DOI:** 10.3389/fonc.2021.814396

**Published:** 2021-12-21

**Authors:** Meng Meng, Tianjun Lan, Duanqing Tian, Zeman Qin, Yu Li, Jinsong Li, Haotian Cao

**Affiliations:** ^1^ Research Center of Medicine, Guangdong Provincial Key Laboratory of Malignant Tumor Epigenetics and Gene Regulation, Sun Yat-sen Memorial Hospital, Sun Yat-sen University, Guangzhou, China; ^2^ National Supercomputer Center in Guangzhou, Sun Yat-sen University, Guangzhou, China; ^3^ Department of Oral & Maxillofacial Surgery, Sun Yat-sen Memorial Hospital, Sun Yat-sen University, Guangzhou, China; ^4^ Department of Stomatology, Sun Yat-sen Memorial Hospital, Sun Yat-sen University, Guangzhou, China; ^5^ Department of Laboratory Medicine, Peking University Shenzhen Hospital, Shenzhen, China

**Keywords:** carcinogenesis, chromatin, clear cell renal cell carcinoma, computational biology, gene expression profiling, weighted gene co-expression network analysis

## Abstract

Clear cell renal cell carcinoma (ccRCC) accounts for 75%–85% of renal cell carcinoma (RCC) and has a poor 5-year survival rate. In recent years, medical advancement has promoted the understanding of the histopathological and molecular characterization of ccRCC; however, the carcinogenesis and molecular mechanisms of ccRCC remain unclear. Chromatin accessibility is an essential determinant of cellular phenotype. This study aimed to explore the potential role of chromatin accessibility in the development and progression of ccRCC. By the combination of open-access genome-wide chromatin accessibility profiles and gene expression profiles in ccRCC, we obtained a total of 13,474 crucial peaks, corresponding to 5,120 crucial genes and 9,185 differentially expressed genes. Moreover, two potential function modules (P2 and G4) that contained 129 upregulated genes were identified *via* the weighted gene co-expression network analysis (WGCNA). Furthermore, we obtained five independent predictors (*FSCN1*, *SLC17A9*, *ANKRD13B*, *ADCY2*, and *MAPT*), and a prognostic model was established based on these genes through the least absolute shrinkage and selection operator-proportional hazards model (LASSO-Cox) analysis. This model can stratify the ccRCC samples into a high-risk and a low-risk group, from which the patients have distinct prognosis. Further analysis demonstrated a completely different immune cell infiltration pattern between these two risk groups. This study also suggested that mast cell resting is associated with the prognosis of ccRCC and could be a target of immunotherapy. Overall, this study indicated that chromatin accessibility plays an essential role in ccRCC. The five prognostic chromatin accessibility biomarkers and the prognostic immune cells can provide a new direction for the treatment of ccRCC.

## Introduction

Renal cell carcinoma (RCC) is among the top 10 most common lethal malignant renal tumors in adults, and clear cell renal cell carcinoma (ccRCC) accounts for 75%–85% of RCC (1). Most ccRCC cases are resistant to chemotherapy and radiotherapy, most likely due to their complicated molecular mechanisms. Surgical excision is still the main treatment. Metastasis occurs in 30% of patients leading to poor 5-year survival rates (2). Early identification and surgical treatment are beneficial for an optimistic prognosis. However, the underlying molecular mechanisms of ccRCC remain ambiguous and effective therapeutic targets are lacking. Therefore, it is urgent to investigate the molecular mechanism and identify genes that are responsible for the carcinogenesis of ccRCC.

DNA is a dynamic structure containing various genetic information, which is packaged into chromatin before it can be stored in the nucleus. Chromatin regulation by chemical modification of DNA or histones modulates gene expression and is important for development, differentiation, and treatment response of cancers. During the process of gene expression, chromatin accessibility plays a crucial role *via* influencing the interaction between genes and their targets. A previous study found that some oncogenes (*BRCA1* and *MYC*) are located in vulnerable regions of chromosomes (2–4). Oncogenes are the main factors responsible for the occurrence and development of tumors. Therefore, exploring the relationship between chromatin accessibility and oncogenes and their role in ccRCC is helpful to clarify the underlying mechanisms. The genomic locations of open chromatin can be uncovered by assay for transposase-accessible chromatin using sequencing (ATAC-seq). ATAC-seq is a high-throughput, sensitive, and rapid method for separately assaying genome-wide chromatin accessibility (open chromatin). ATAC-seq made it possible to assess the gene regulatory landscape in primary human cancers, especially the oncogenes that are usually upregulated in tumors (5).

This study aimed to identify the key chromatin accessibility genes of ccRCC using the combination of open-access genome-wide chromatin accessibility profiles, gene expression profiles, and protein–protein interactions. Additionally, we evaluated the prognostic value of these selected gene biomarkers in clear cell renal cell carcinoma.

## Materials and Methods

### Data Acquisition

The open-access genome-wide chromatin accessibility profiles (ATAC-seq) containing 94,864 peakCalls generated from 16 ccRCC tissues were downloaded from the NCI Genomic Data Commons (https://gdc.cancer.gov/) in The Cancer Genome Atlas (TCGA). The RNA-sequencing (RNA-seq) data of the above 16 ccRCC patients were also included in the study. Moreover, after removing the samples without complete clinical data, we extracted the transcription data and clinical follow-up information of 250 ccRCC samples and 40 normal tissues from the UCSC Xena dataset (https://xenabrowser.net/) for further analysis.

### Selection of Crucial Peaks and Genes

In the present study, we used edge package (http://bioconductor.org/packages/edgeR/) to normalize and calculate counts per million (CPM) with parameters (matrix = counts, log = TRUE, prior.count = 5) for ATAC-seq and RNA-seq data, respectively. Then, the correlation coefficient between ATAC-seq peak count and RNA-seq gene count was calculated. The peaks and corresponding genes with a correlation coefficient ≥0.6 and *p*-value ≤0.05 were selected as crucial biomarkers. We applied the ChIPseeker (version 1.22.1) software (6, 7) to annotate all crucial peakCalls *via* the symbol (hg38) using R (version 3.6.3, Windows x64 bit).

### Identification of Differentially Expressed Genes

We used linear models for microarray data package (limma http://www.bioconductor.org/packages/release/bioc/html/limma.html) to identify the differentially expressed genes (DEGs) with a correlation coefficient ≥0.6 from 40 normal tissues and 250 ccRCC samples. Adjust *p*-value ≤0.05 and |logFC| ≥1were defined as the cutoff standards (8).

### Weighted Correlation Network Analysis for the Crucial Peaks and Genes

Weighted gene co-expression network analysis (WGCNA) is a method to analyze gene expression patterns of multiple samples, which can cluster genes with similar expression patterns and analyze the association between modules and specific traits or phenotypes (9, 10). First, we applied the WGCNA analysis for the crucial peaks to investigate the expression pattern, with power = 8, maxBlockSize = 15,000, TOMType = “unsigned,” minModuleSize = 30, reassignThreshold = 0, and mergeCutHeight = 0.25. Similarly, we carried out WGCNA analysis in the genes correlated to these peaks. Then, the modules were divided into several groups based on the cluster tree in peaks and genes. Next, the group with the largest number of crucial peaks and correlation DEGs and the group with the largest number of crucial genes and DEGs were selected. Lastly, the intersection among upregulated genes and the two groups was identified as hub genes.

### Functional Enrichment Analysis and Protein–Protein Interaction Network Construction

Gene Ontology (GO) analysis and Kyoto Encyclopedia of Gene and Genomes (KEGG) pathway enrichment were used to understand the molecular function and pathway of hub genes *via* the enrichGO and enrichKEGG packages (11). The Search Tool for the Retrieval of Interacting Genes (STRING) was used to construct the protein–protein interaction (PPI) network for these hub genes, and the results were visualized by Cytoscape software (12). The node and edge of the PPI network were obtained based on the score >0.400 (medium confidence).

### Construction of the Prognostic Model

In this study, the univariate Cox regression analysis and Kaplan–Meier test were applied to screen candidate prognosis-related genes. Then, we applied the least absolute shrinkage and selection operator (LASSO) analysis to further refine the prognosis-related genes (13). Finally, we constructed a multiple stepwise Cox regression model based on these selected genes, and the formula for the risk score was as follows:


Risk score=Exp1β1+Exp2β2+Exp3β3+⋯

*Exp* represents the expression level of the gene and β represents the coefficient.

Based on the model, a risk score was calculated for each sample and these cases were divided into the high-risk and low-risk groups. In order to assess the ability of the model for predicting OS, the time-dependent receiver operating characteristic (ROC) curve analysis and the Harrell’s concordance index (C-index) were used in the study. The area under the curve at 1, 3, and 5 years was used to quantify the predictive ability of the model. The principal component analysis (PCA) and t-distributed stochastic neighbor embedding (t-SNE) analysis were used to explore the distribution of different groups based on the expression level of genes. Moreover, we performed the univariate and multiple Cox regression analyses to evaluate the association between clinical factors and prognoses.

### Immune Cell Infiltration Analysis

In order to understand the immune cell infiltration pattern of ccRCC, we performed CIBERSORT analysis on the high- and low-risk groups to quantify the composition of 22 types of immune cells in the tumor (14). Then, we analyzed the relationship between immune cells and risk scores to select the risk-related immune cells. Finally, we use Kaplan–Meier analysis to screen the immune cells related to prognosis.

## Results

### Selection of Crucial Peaks and Genes and Identification of DEGs

**Figure 1:**
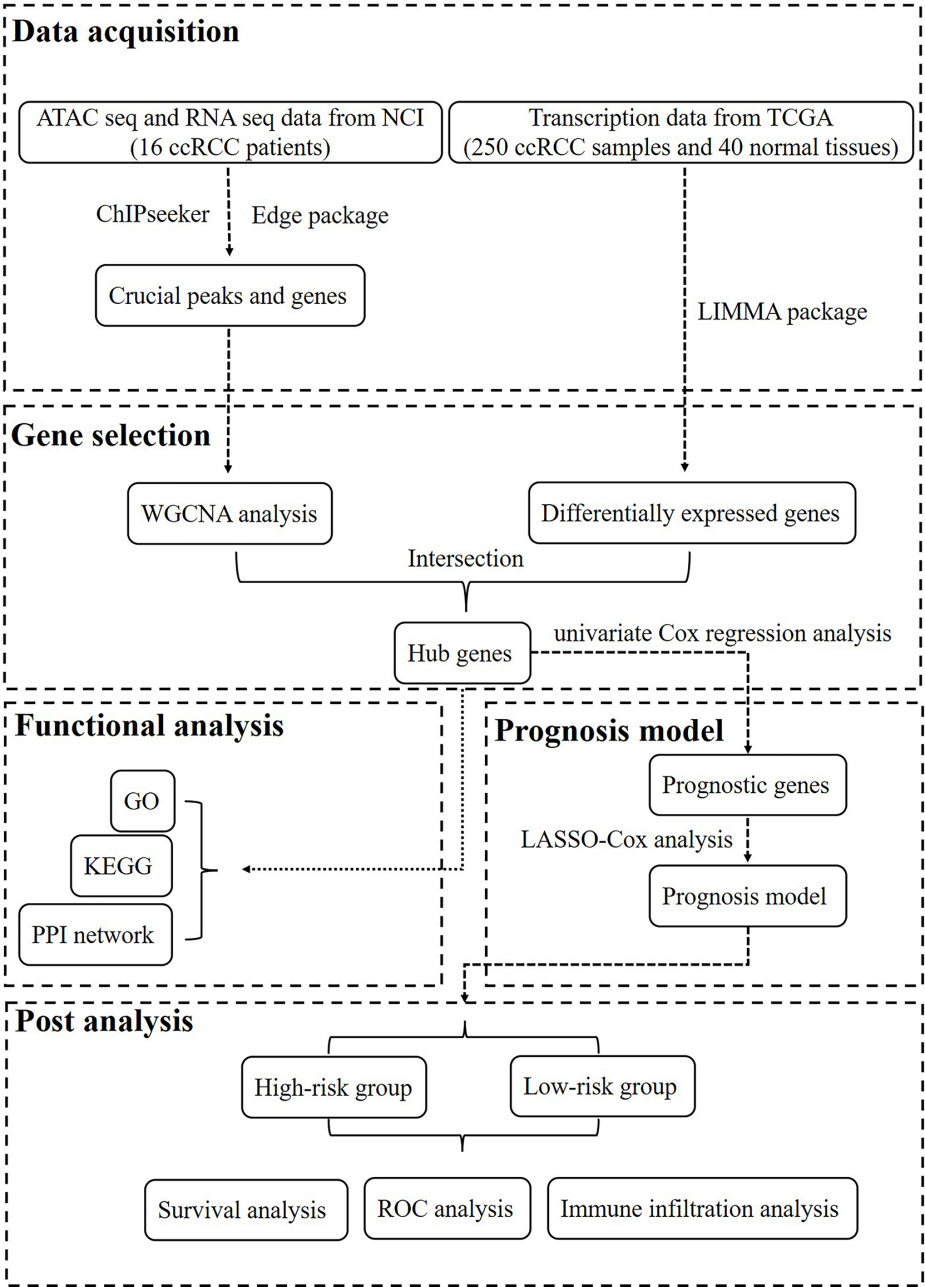
The workflow of this study.

**Figure 2:**
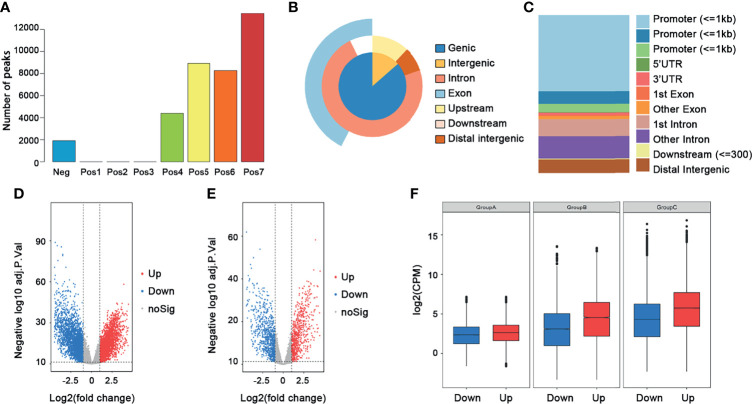
Selection of crucial peaks and genes. **(A)** The distribution of correlation coefficient between peaks and genes. Neg, correlation coefficient ≤0; Pos1, 0 ≤ correlation coefficient ≤ 0.1; Pos2, 0.1 ≤ correlation coefficient ≤ 0.2; Pos3, 0.2 ≤ correlation coefficient ≤ 0.3; Pos4, 0.3 ≤ correlation coefficient ≤ 0.4; Pos5, 0.4 ≤ correlation coefficient ≤ 0.5; Pos6, 0.5 ≤ correlation coefficient ≤ 0.6; Pos7, 0.6 ≤ correlation coefficient ≤ 1. **(B)** The location distribution of crucial peaks in the genome. **(C)** The location distribution of crucial genes in the genome. **(D)** The volcano plot of the DEGs between 250 tumor tissues and 40 normal tissues. Red, upregulated genes; blue, downregulated genes. **(E)** The volcano plot of the differentially expressed crucial genes between 250 tumors and 40 normal tissues. Red, upregulated genes; blue, downregulated genes. **(F)** The distribution of DEGs among ATAC-seq data, RNA-seq data, and transcription data. Group A, ATAC-seq data; group B, RNA-seq data; group C, transcription data.

The workflow of this study is shown in **Figure 1**. In total, 250 ccRCC cases and 40 normal samples were included in our study, and the clinical information of the ccRCC patients is shown in **Table 1**. First, we calculated the correlation coefficient between ATAC-seq peak count and RNA-seq gene count. A total of 13,474 crucial peaks with correlation coefficient ≥0.6 and *p*-value ≤0.05 and the corresponding 5,120 crucial genes were selected (**Figure 2**). **Figures 2B, C** present the location distribution of the 13,474 crucial peaks in the genome. Most of the crucial peaks were found located on the regions of promoters. Through the limma package, we identified a total of 9,185 DEGs between tumor and normal tissues, including 4,933 upregulated genes and 4,251 downregulated genes (**Figure 2D**). Then, for the 5,120 crucial genes, 740 upregulated genes and 932 downregulated genes were screened out (**Figure 2E**). As shown in **Figure 2F**, the gene expression and chromatin accessibility of DEGs was consistent. The bar plots displayed in groups A, B, and C present the peak count of DEGs from 16 ccRCC samples, the gene count of DEGs from 16 ccRCC samples, and the gene count of DEGs from 250 ccRCC samples, respectively. Compared with the downregulated genes, the upregulated genes have higher peak counts, which represented to be easily bound by transcription factors (**Figure 2F**).

**Table 1:** The clinical characteristics of the patients with ccRCC.

Variables	ATAC-seq	RNA-seq
***n***	16	250
**Age**		
<60	5	106
≥60	11	144
**Gender**		
Female	7	98
Male	9	152
**Grade**		
Grade 1	1	3
Grade 2	6	106
Grade 3	4	100
Grade 4	3	37
Unknown	2	4
**Stage**		
**T**		
1	9	106
2	4	42
3	3	95
4	0	7
**N**		
0	3	235
1	0	15
Unknown	13	0
**M**		
0	4	209
1	1	41
Unknown	11	0
**OS days (average)**	760.06	1,362.72

### WGCNA for Crucial Peaks and Genes

**Figure 3:**
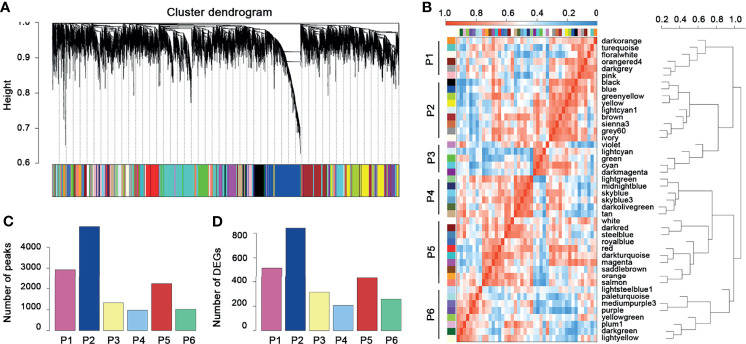
WGCNA for the crucial peaks. **(A)** Cluster dendrogram of crucial peaks. **(B)** Module–module correlation heatmap of 44 modules in crucial peaks. Red, high correlation; blue, low correlation. **(C)** The distribution of crucial peaks among six groups. **(D)** The distribution of DEGs and corresponding crucial peaks among six groups.

**Figure 4:**
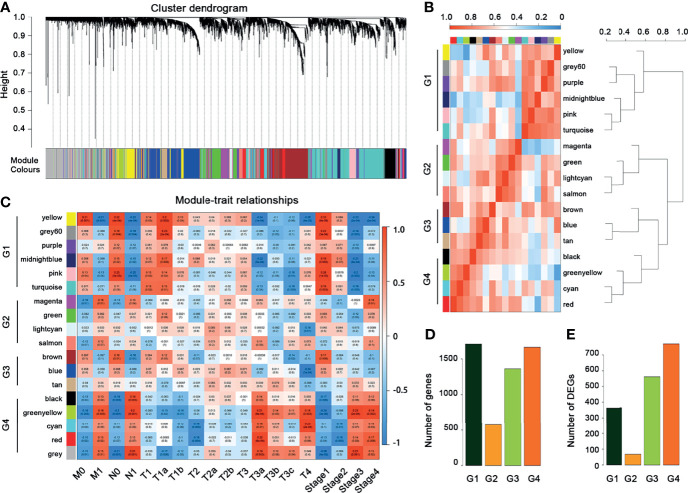
WGCNA for the crucial genes. **(A)** Cluster dendrogram of crucial genes. **(B)** Module–module correlation heatmap of 17 modules in crucial genes. Red, high correlation; blue, low correlation. **(C)** Relationships between mRNA modules and clinical characteristics. **(D)** The distribution of crucial genes among four groups. **(E)** The distribution of DEGs among four groups.

**Figure 5:**
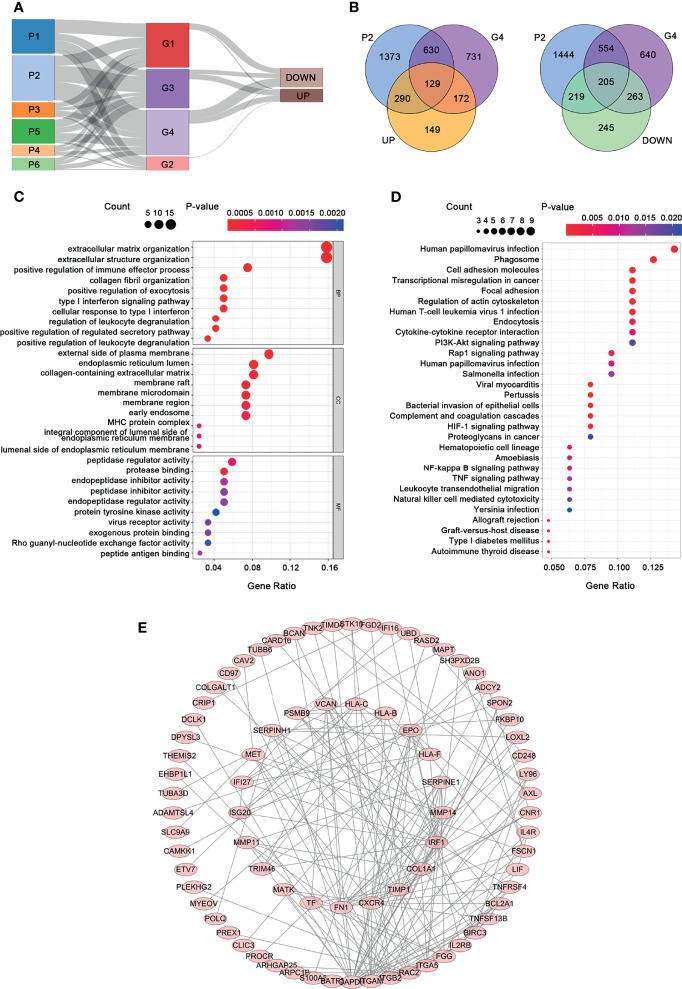
Functional analysis and PPI construction of upregulated genes. **(A)** The Sankey plot. **(B)** The Venn diagram. **(C)** GO analysis. **(D)** KEGG pathway analysis. **(E)** PPI network.

The 13,474 crucial peaks were selected for further WGCNA analysis. In this study, we set 8 as the threshold power to obtain a high average connectivity degree and a cluster dendrogram was clustered according to the power (**Figure 3A**). In the cluster dendrogram, 44 color modules were selected among crucial peaks and divided into six groups based on the correlation among modules (**Figure 3B**). Among the six groups, the P2 group contains the greatest number of peaks and DEGs (**Figures 3C, D** **)**. Similarly, we applied the WGCNA for the 5,120 crucial genes and set the power as 7. We obtained 17 modules and these modules were divided into four groups (**Figures 4A, B** **)**. The genes in these 17 modules were selected to further analyze the relationship between traits (clinical stages) and modules (**Figure 4C**). The G4 group was associated with worse clinical status, while the G1 group was associated with better clinical status. Moreover, the G4 group contains the largest number of peaks and DEGs (**Figures 4D, E** **)**. Therefore, we considered that the DEGs in P2 and G4 may play potential roles in ccRCC. We used the Sankey plot and Venn diagram to investigate the overlap of DEGs in the P2 and G4 groups (**Figures 5A, B** **)**. Lastly, 129 upregulated genes and 205 downregulated genes were found in the G4 and P2 groups (**Figure 5B**). The 129 upregulated genes were selected for further analysis.

### Functional Analysis and PPI Network Construction

GO functional and KEGG pathway enrichment analyses for the 129 upregulated genes are presented in **Figures 5C, D**. A significant enrichment in the biological process was found in related to extracellular matrix, such as extracellular matrix organization, extracellular structure organization, and collagen fibril organization (**Figure 5C**, *p* < 0.05). As for the cell component tests, the upregulated genes were enriched in the construction of the membrane, such as the external side of the plasma membrane, endoplasmic reticulum lumen, membrane raft, membrane microdomain, and membrane region. In addition, these upregulated genes are mainly related to protein regulation in molecular function, including protease binding, peptidase regulator activity, peptide antigen binding, endopeptidase inhibitor activity, and endopeptidase regulator activity. KEGG pathway analysis presented that the upregulated genes were significantly enriched in human papillomavirus infection, phagosome, cell adhesion molecules, and so on. In order to understand the relationship among the upregulated genes, a PPI network was constructed based on these 129 differently expressed oncogenes. The PPI network contained a total of 85 nodes and 201 edges (**Figure 5E**) that represent the hub genes and pathways, respectively.

### Construction of the Prognostic Model

**Figure 6:**
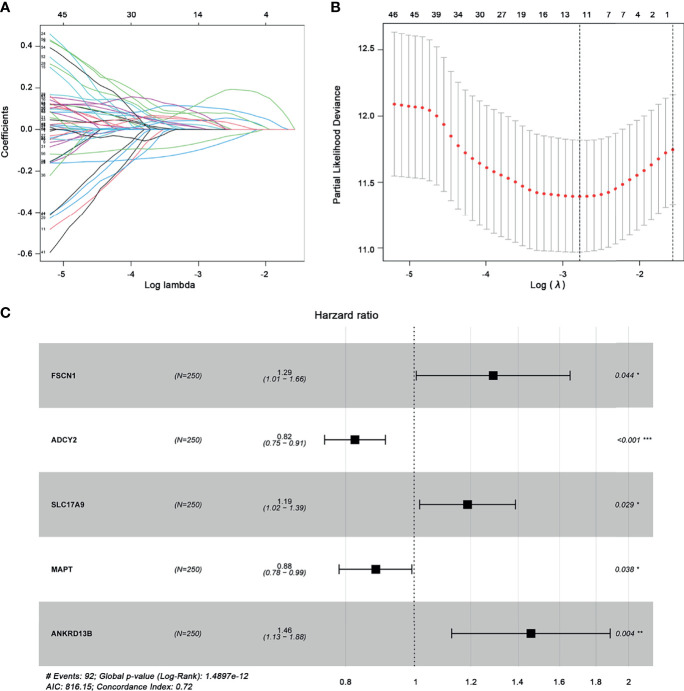
Construction of the prognostic gene model. **(A)** Selection of optimal parameter (lambda) in LASSO analysis. **(B)** LASSO coefficient profiles of the 11 prognostic genes. **(C)** Multivariate Cox regression analysis determines the five prognostic genes as independent predictors in ccRCC. *p < 0.05; **p < 0.01, ***p < 0.001.

Through univariate Cox regression analysis, 62 prognostic genes with a *p*-value less than 0.05 were obtained. Further LASSO analysis selected 11 genes as potential prognostic markers. **Figure 6A** shows that the lowest lambda value can be obtained when 11 genes are involved. **Figure 6B** shows the LASSO regression coefficient curve of 11 genes. Then, these 11 genes were analyzed by multiple stepwise Cox regression model and five genes (*FSCN1*, *SLC17A9*, *ANKRD13B*, *ADCY2*, and *MAPT*) were finally used as independent predictors to construct the prognostic model (**Figure 6C**). The model is as follows:


$$\begin{array}{c} \text{Risk score}=0.2555\text{ExpFSCN}1+0.1725\text{ExpSLC}17\text{A}9+0.3769\text{ExpANKRD}13\text{B}-0.1930\text{ExpADCY}2\\ -0.1259\text{ExpMAPT} \end{array}$$

**Figure 7:**
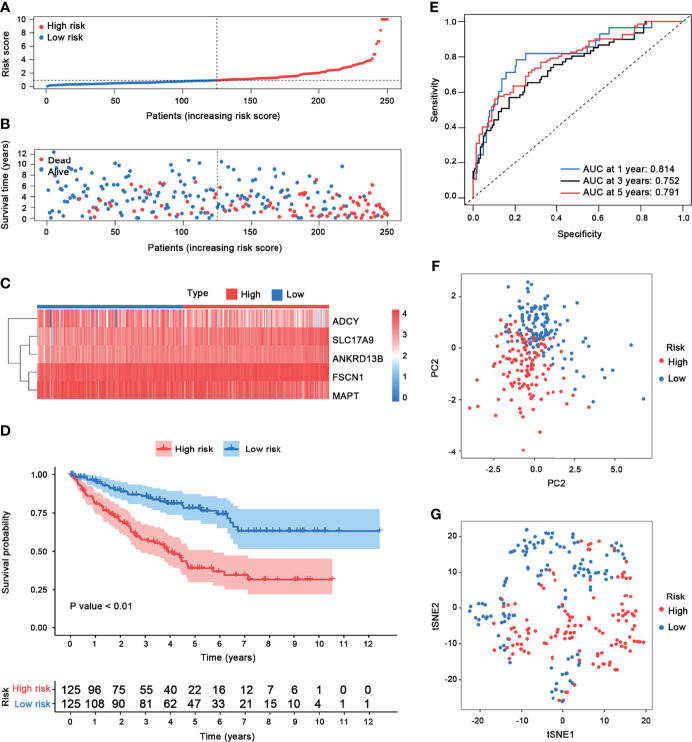
Risk score analysis. **(A–C)** Gene model risk score distribution, survival times and status, and heatmap of the expression of five prognostic genes in patients with ccRCC. The samples were classified into the low-risk and the high-risk groups based on the cutoff value of risk scores. **(D)** Kaplan–Meier curves of OS for patients with ccRCC based on the prognostic model. **(E)** Time-dependent ROC curves of the prognostic model. **(F)** PCA plot. **(G)** t-SNE analysis.

**Figure 8:**
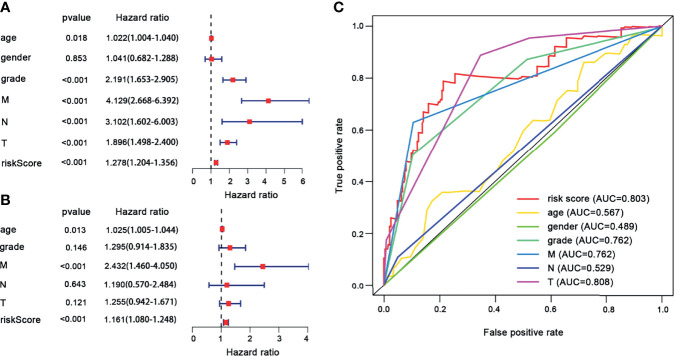
The univariate and multivariate Cox regression analyses. **(A)** Univariate Cox regression analyses. **(B)** Multivariate Cox regression analyses. **(C)** ROC curves of the risk score and clinical characteristics for 1-year survival.

According to this prognostic model, we found that *FSCN1*, *SLC17A9*, and *ANKRD13B* are oncogenes, and overexpression of these genes represents a poor prognosis, whereas *ADCY2* and *MAPT* are tumor suppressor genes and associated with a better prognosis of ccRCC. Based on the median risk score, ccRCC patients were divided into the high-risk and the low-risk groups (**Figure 7A–C**). As shown in **Figure 7D**, Kaplan–Meier survival analysis and log-rank test were used to compare the differences in OS between the high-risk group and the low-risk group. The results showed that the prognosis of patients in the high-risk group was worse than that in the low-risk group. In addition, the values of the area under the curve of the ROC curve (AUC) of 1-, 3-, and 5-year survival rates were 0.814, 0.752, and 0.791, respectively, indicating that the prediction accuracy of this prognostic model was superior (**Figure 7E**). PCA and t-SNE analysis showed that patients in different risk groups were distributed in two directions, which represented different gene expression patterns between the two groups (**Figures 7F, G** **)**. Then, we conducted univariate and multivariate Cox regression analyses to explore whether the genes selected in this study can be used as independent prognostic factors of ccRCC. Univariate analysis showed that risk score (HR = 1.278, 95% CI = 1.204–1.356, *p* < 0.001), age (HR = 1.022, 95% CI = 1.004–1.040, *p* = 0.018), grade (HR = 2.191, 95% CI = 1.653–2.905, *p* < 0.001), M stage (HR = 4.129, 95% CI = 2.668–6.392, *p* ≤ 0.001), N stage (HR = 3.102, 95% CI = 1.602–6.003, *p* < 0.001), and T stage (HR = 1.896, 95% CI = 1.498–2.400, *p* < 0.001) were significantly correlated with the prognosis of ccRCC patients (**Figure 8A**). In addition, after adjusting for clinical characteristics such as age, grade, T stage, M stage, and N stage, the risk score was still an independent predictor of ccRCC patients in multivariate analysis (HR = 1.161, 95% CI = 1.080–1.248, *p* < 0.001) (**Figure 8B**). **Figure 8C** shows the superior predictive performance of this gene model (AUC = 0.803) than age (AUC = 0.567), gender (AUC = 0.489), grade (AUC = 0.762), M staging (AUC = 0.762), and N staging (AUC = 0.529).

### Immune Cell Infiltration Between the High-Risk and the Low-Risk Groups

**Figure 9:**
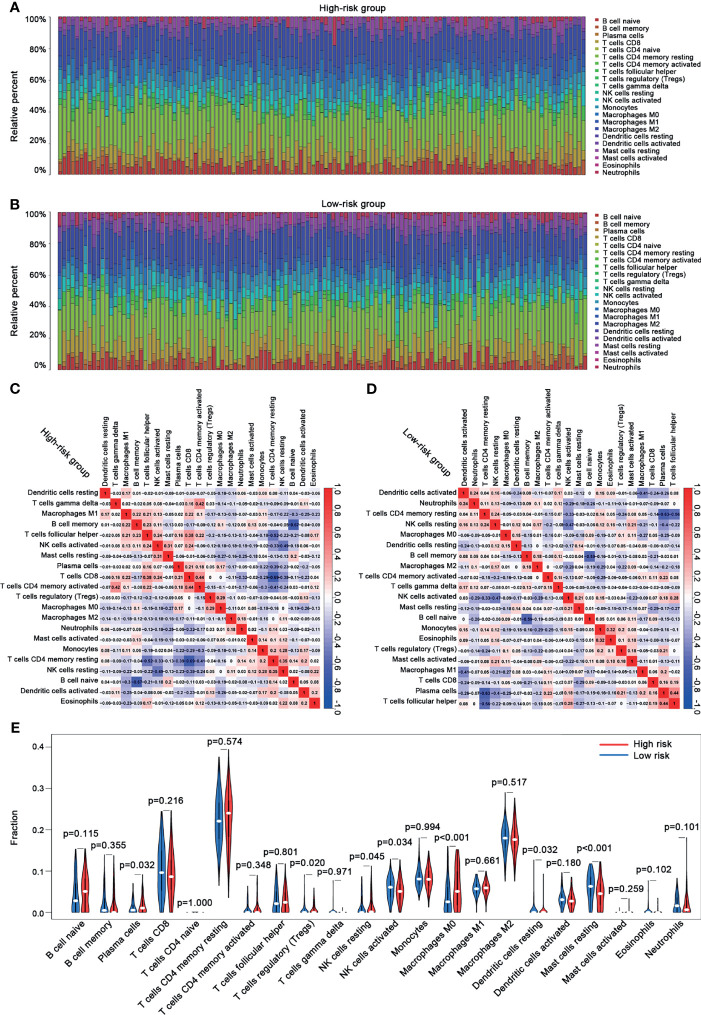
The immune cell infiltration analysis between the high-risk and the low-risk groups. **(A)** The bar chart of the proportion of immune cells in the high-risk group. **(B)** The bar chart of the proportion of immune cells in the low-risk group. **(C)** The correlation analysis between immune cells and risk scores in the high-risk group. **(D)** The correlation analysis between immune cells and risk scores in the low-risk group. **(E)** Violin plots showing the different abundance of the immune cells between the high-risk (red) and the low-risk (green) groups.

**Figure 10:**
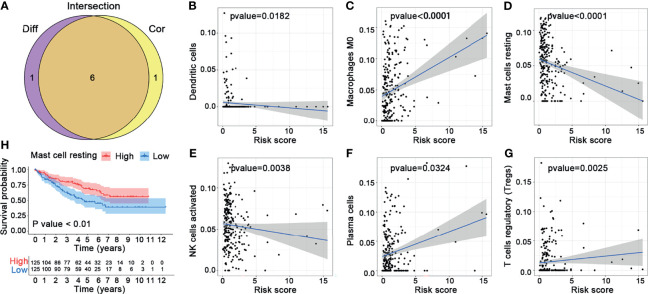
Comprehensive analysis for the immune cells. **(A)** Venn diagram. **(B–G)** Correlation analysis between the six hub immune cells and risk scores. **(H)** Kaplan–Meier analysis of mast cells resting.

In this study, we evaluated the composition of 22 types of immune cells in each tumor sample and compared the immune cell infiltration patterns between the high-risk and the low-risk groups (**Figure 9**). As shown in **Figures 9C, D**, the pattern of immune cell infiltration is completely different between the high-risk and the low-risk groups, suggesting that the immune system played a corresponding role in part through the five genes that build the model. In addition, we found that there were significant differences in plasma cells, Tregs, NK cells resting, NK cells activated, macrophages M0, dendritic cells resting, and mast cells resting between the high-risk and the low-risk groups. Plasma cells, Tregs, NK cells resting, and macrophages M0 increased significantly in the high-risk group, while NK cells activated, dendritic cells resting, and mast cells resting increased in the low-risk group (**Figure 9E**). Then, through the correlation analysis, we found that seven types of immune cells were related to the risk score (plasma cells, Tregs, NK cells activated, macrophages M0, dendritic cells resting, mast cells resting, and eosinophils) (**Table 2**). Next, we obtained six hub immune cells *via* Venn diagram analysis (**Figures 10A–G**). To further analyze the role of these cells for the prognosis of ccRCC, we performed Kaplan–Meier analysis. Finally, we found that mast cells resting are associated with prognosis, which could be a target of immunotherapy (**Figure 10H**).

**Table 2:** The correlation between immune cells and risk scores.

Cells	*R*	*p*-value
Plasma cells	0.14	0.032
T cells regulatory	0.19	0.0025
NK cells activated	−0.18	0.0038
Macrophages M0	0.28	5.7e-6
Dendritic cell resting	−0.15	0.018
Mast cell resting	−0.3	1.8e-6
Eosinophils	−0.14	0.025

## Discussion

Because of the poor prognosis of ccRCC, it is critical to understand the molecular mechanisms leading to its development and progression. In recent years, multi-omics analysis methods emerged and are often used in the study of hub genes in various cancers. For example, high-throughput data such as large-scale gene expression profiles and protein–protein interactions are used to screen hub genes in different diseases. Integrated bioinformatics analysis and multi-omics analysis for diseases and cancers enrich our knowledge of important genes and pathways and provide new insights into their functions and mechanisms. These methods may aid in the identification of target genes of interest for the diagnosis and treatment of ccRCC.

Previous studies have used bioinformatics to investigate genes that affect the survival of patients with ccRCC. However, researchers obtained different results by using different bioinformatics analytic methods when they tried to identify the key genes of the same disease. Wei et al. reported that the expression of certain hub genes (*CDKN3*, *TPX2*, *BUB1B*, *CDCA8*, *UBE2C*, *NDC80*, *RRM2*, *NCAPG*, *NCAPH*, *PTTG1*, *FAM64A*, *ANLN*, *KIF4A*, *CEP55*, *CENPF*, *KIF20A*, *ASPM*, and *HJURP*) was closely associated with overall survival and recurrence-free survival of patients with ccRCC using KEGG pathway analysis and PPI network construction (15). Similarly, Yuan et al. identified 10 hub genes (*TOP2A*, *MYC*, *ALB*, *CDK1*, *VEGFA*, *MMP9*, *PTPRC*, *CASR*, *EGFR*, and *PTGS2*) and six ccRCC-related modules by using co-expression analysis and PPI network construction (2). Different from the previous studies, we introduced the analysis of ATAC-seq and RNA-seq to detect the correlation between genes of chromatin accessibility and gene expression patterns. Then, WGCNA analysis was applied to identify the peak and gene modules associated with clinical stages. Lastly, we used LASSO-Cox analysis to further refine the prognosis-related genes in the study.

By calculating the correlation coefficient between ATAC-seq peak count and RNA-seq gene count, we identified 13,474 crucial peaks and the corresponding 5,120 crucial genes (correlation coefficient ≥ 0.6; *p*-value ≤ 0.05). Then, we used the WGCNA method to analyze the potential expression pattern in crucial peaks and crucial genes. The P2 group and the G4 group were screened as potentially functional modules for ccRCC and contained 129 upregulated genes that were defined as potential oncogenes. Among the 129 oncogenes, 62 genes were confirmed to be associated with the prognosis of ccRCC. After the LASSO-Cox analysis, we identified five independent predictors (*FSCN1*, *SLC17A9*, *ANKRD13B*, *ADCY2*, and *MAPT*) to construct a prognostic model, which can recognize the genome difference among patients with ccRCC and divide these patients into the high-risk and the low-risk groups. Moreover, this model has a better performance than other clinical factors. Thus, our study provides a reliable and accurate predictive method for the prognosis of ccRCC patients.

In the GO and KEGG analyses, we found that the upregulated genes were significantly enriched in the extracellular matrix and human papillomavirus infection. As CAF-specific mediators, remodeling of the ECM and immunomodulation affects the cellular and structural architecture of each tissue in cancers (16). ECM-associated macromolecules govern a wide range of cell functions such as migration, invasion, adhesion, and differentiation and are key players in immunopathology, chronic inflammatory disease, tissue fibrosis, and cancer. The association between ccRCC and HPV infection has not been well studied. (17) reported that HPV infection, especially the high-risk type of HIV, is associated with ccRCC. Simultaneously, some research reported that immunotherapy allows a significant benefit in improving efficacy and safety in patients with HPV-positive anal cancer and head and neck cancers.

Among the five independent predictor genes, *FSCN1* and *SLC17A9* were highly expressed in various tumors. Overexpression of *FSCN1* and *SLC17A9* predicted a poor prognosis (18). *FSCN1* is a member of the fascin family of actin-binding proteins and plays a critical role in motility, adhesion, and cell migration. In ccRCC, *FSCN1* is an oncogene that is regulated by the PI3K/AKT signaling pathway. The PI3K/AKT inhibitors or knockdown GSK-3β decreased *FSCN1* expression in ccRCC cells and attenuated ccRCC cell invasion. *SLC17A9*, a transmembrane protein, participates in the vesicular uptake, storage, and secretion of adenosine triphosphate (ATP). High *SLC17A9* expression correlates with poor survival in hepatocellular carcinoma, gastric carcinoma, and colorectal cancer. However, there is no research on the role of *SLC17A9* in ccRCC. In the study, we found that *SLC17A9* may be an oncogene and represent a poor prognosis in ccRCC. The role of *ANKRD13* in cancers is poorly studied, with only three articles on this family gene reported in PubMed (19). The *ANKRD13* family proteins contain ubiquitin-integrating motifs that recognize the Lys-63-linked ubiquitin chains appended to EGFR after ligand biding. Nam-Yun Cho et al. identified *ANKRD13* as one of the eight cancer-specific methylated loci in colorectal cancer using cancer-specific DNA methylation markers. *ADCY2* is known to associate with bipolar disorder and severe chronic obstructive pulmonary disease (20). Aberrant methylation of *ADCY2* is observed in oral cancer, colorectal cancer, prostate cancer, and urinary bladder cancer. In our study, *ADCY2* presents as a potential suppressor gene with better prognosis. The *MAPT* gene is involved in dozens of neurodegenerative diseases called tauopathies, including frontotemporal lobar degeneration/frontotemporal dementia (FTLD/FTD) and Alzheimer’s disease (AD) (21). Sun et al. reported that low *MAPT* expression is associated with poor OS and DSS and shorter PFI in ccRCC (21). These five biomarkers of chromatin accessibility may provide potential therapeutic targets for ccRCC.

Recently, multiple lines of evidence indicate that quantification of immune cell infiltrates can predict the outcomes of patients with ccRCC. The tumor microenvironment (TME) is composed of stromal cells, immune cells, extracellular matrix molecules, and inflammatory mediators (22). Cancer cells exploit the mechanisms that immune checkpoint receptors may inhibit the activity of killer and proinflammatory lymphocytes, following binding to specific ligands, to inactivate tumor-infiltrating lymphocytes (TILs) in order to escape from immunosurveillance and survive (23). In the present study, we performed a comprehensive analysis on the relationship between immune cells and chromatin accessibility markers between the two distinct risk groups. Through CIBERSORT analysis (14), we found that the pattern of immune cell infiltration is completely different between the high-risk and the low-risk groups, suggesting that the immune system plays a corresponding role in part through the five genes that we used to build the prognostic model. A previous study suggested that CD8^+^ TILs and PD1^+^ TILs have a co-stimulatory effect and a high density of CD8^+^ TILs is associated with poor clinical outcome (24). In our study, we found that mast cells resting are associated with good prognosis, which may be a target of immunotherapy. Chen et al. reported that infiltrating mast cells promote renal cell carcinoma angiogenesis by modulating the PI3K–AKT–GSK-3β–AM signaling pathway (25). Some similar results were also found in triple-negative breast cancer and head and neck cancer.

The limitation of this study is that normal tissue samples were absent in the ATAC-seq data of the 16 ccRCC patients from TCGA used in the study. More reliable and significant discoveries might be obtained if we can combine ATAC-seq data from normal tissue samples. Also, the results would be more reliable if the study included clinical and basic experimental data to support the results.

## Conclusion

In conclusion, our study indicated that chromatin accessibility plays a role in ccRCC. We identified five prognostic biomarkers of chromatin accessibility (*FSCN1*, *SLC17A9*, *ANKRD13B*, *ADCY2*, and *MAPT*) and constructed a prognostic model based on these five biomarkers. This model can predict the prognosis of ccRCC. In addition, we found that mast cells resting are associated with the prognosis of ccRCC and could be a target of immunotherapy.

## Data Availability Statement

The ATAC-seq and RNA-seq data that support the results of this study are available at the TCGA repository (https://portal.gdc.cancer.gov/).

## Author Contributions

HC conceived and designed the research. MM, TL, and YL analyzed the data. JL participated in the design of some bioinformatics analysis. MM and TL wrote the manuscript. JL, DT, ZQ, and HC edited the manuscript.

## Funding

This research was supported by the National Natural Science Foundation of China (81903069) and the China Postdoctoral Science Foundation (2019M663292, 2019M663315).

## Conflict of Interest

The authors declare that the research was conducted in the absence of any commercial or financial relationships that could be construed as a potential conflict of interest.

## Publisher’s Note

All claims expressed in this article are solely those of the authors and do not necessarily represent those of their affiliated organizations, or those of the publisher, the editors and the reviewers. Any product that may be evaluated in this article, or claim that may be made by its manufacturer, is not guaranteed or endorsed by the publisher.
